# Second line chemotherapy and visceral metastases are associated with poor survival in patients with mCRPC receiving ^177^Lu-PSMA-617

**DOI:** 10.7150/thno.35759

**Published:** 2019-07-09

**Authors:** Katharina Kessel, Robert Seifert, Michael Schäfers, Matthias Weckesser, Katrin Schlack, Martin Boegemann, Kambiz Rahbar

**Affiliations:** 1 Department of Nuclear Medicine, Münster University Hospital; 2 Department of Urology, Münster University Hospital

**Keywords:** prostate cancer, mCRPC, ^177^Lu-PSMA-617, PSMA, radioligand therapy, second line chemotherapy, cabazitaxel, metastases

## Abstract

The purpose of this study was to identify previous treatments and biomarker profile features that prognosticate overall survival (OS) in patients with mCRPC receiving ^177^Lu-PSMA-617.

**Methods:** 109 mCRPC patients treated with a median of 3 cycles of ^177^Lu-PSMA-617 were included. Data were analyzed according to OS as well as PSA response patterns with regard to prior therapies, laboratory biomarkers and metastatic extent in univariate as well as multivariate Cox's proportional hazards models. PSA decline was assessed using the lowest PSA levels after the first cycle of therapy (initial PSA response) and during the entire observation period (best PSA response).

**Results:** In total, 54 patients (49.5%) died during the observation period. First and second line chemotherapy were performed in 85% and 26%, and Abiraterone and Enzalutamide were administered in 83% and 85%, respectively. Any initial PSA decline occurred in 55% while 25% showed a PSA decline of ≥50%. The median estimated OS was 9.9 months (95% CI: 7.2-12.5) for all patients. Any initial decline of PSA was associated with significantly prolonged OS (15.5 vs. 5.7 months, *p =* 0.002). Second line cabazitaxel chemotherapy (6.7 *vs.* 15.7 months, *p =* 0.002) and presence of visceral metastases (5.9 *vs.* 16.4 months, *p*<0.001) were associated with shorter OS. Only visceral metastases remained significant in a multivariate analysis.

**Conclusion:** ^177^Lu-PSMA-617 is an effective therapy for patients with mCRPC. However, the present data indicate that its beneficial effects on OS are strongly influenced by pretreatment (history of second line chemotherapy with cabazitaxel) and the presence of visceral metastases at onset of ^177^Lu-PSMA-617 treatment.

## Introduction

Prostate-specific membrane antigen (PSMA) ligands are used both for diagnostics and therapy of patients with prostate cancer and are indeed changing the management of prostate cancer patients 1-4.

The most commonly used radiopharmaceutical for patients with advanced metastatic castration- resistant prostate cancer (mCRPC), ^177^Lu-labelled PSMA-617, has been the subject of various studies during recent years 3, 5-12. The advantages of treatment with ^177^Lu-PSMA-617 are the reported low toxicity profile, the high response rates and an improvement in quality of life, even in comparison to other third-line treatments like Cabazitaxel and Enzalutamide 13. Retrospective studies showed promising response rates indicating the capability to improve clinical endpoints like progression-free survival and PSA response 6, 12, 14. Moreover, it was shown that ^177^Lu‑PSMA‑617 therapy may even prolong overall survival (OS) in a heavily pretreated patient cohort. In a previous study, an initial PSA decline of 20.9% after the first ^177^Lu-PSMA-617 cycle was independently associated with longer OS 15. More reliable markers and predictors are urgently needed to stratify patients for upcoming randomized controlled trials on ^177^Lu-PSMA-617 and select for optimized therapy sequencing in mCRPC.

The aim of the present study was to identify factors in addition to PSA response that may help prognosticating OS in patients treated with ^177^Lu-PSMA-617. To this end, the impact of earlier therapies, such as second-line chemotherapy with Cabazitaxel, are analyzed to further elucidate the factors determining the success of ^177^Lu-PSMA-617 therapy. It is known that visceral metastases of patients with mCRPC are associated with poor outcome 16. Especially liver metastases were shown to be associated with shorter OS during different therapies targeting mCRPC 17, 18. For the present initial analysis, the influence of visceral metastases on OS was further investigated in the context of Cabazitaxel treatment in this cohort.

## Patients and Methods

### Patient population

A total of 109 patients treated with ^177^Lu-PSMA- 617 radioligand therapy (RLT) between November 2014 and December 2018 were included in the present retrospective analysis. All patients were castration- resistant and were pretreated with at least one line of chemotherapy (or were not suitable for chemotherapy) as well as at least one of next-generation anti-hormonal therapies (Abiraterone or Enzalutamide). All patients were informed about possible adverse events and risks in detail which may be associated with ^177^Lu-PSMA-617 therapy. All patients gave their written informed consent prior to treatment. The decision for ^177^Lu-PSMA therapy was made by the interdisciplinary tumor board after carefully reviewing each individual case. PSMA-RLT is strictly considered last-line therapy after progression or failure of all other therapy options. 26 patients were included in a previous multicenter study 15. For those patients longer follow-up is now available.

All procedures performed in this study were done in accordance with ethical standards and according to the 1964 Helsinki Declaration and its later amendments. This study was approved by the local ethics committee (No. 2016-585-f-S, Ethikkommission der Ärztekammer Westfalen-Lippe und der Westfälischen Wilhelms-Universität Münster).

### Preparation and administration of ^177^Lu-PSMA-617

The PSMA-617 precursor was purchased from ABX advanced biochemical compounds (Radeberg, Germany) and labelled with ^177^Lutetium (ITG Isotopes Technology, Garching, Germany) on site as previously described. RLT injection was performed as already described elsewhere 15. ^177^Lu-PSMA-617 RLT cycles were administered every 6-8 weeks until tumor progression, death, or withdrawal of the patient's consent.

### Evaluation of response and survival

Overall survival of each patient was determined as the primary clinical endpoint and was defined as death from any cause. PSA decline was assessed using the lowest PSA levels after the first therapy cycle (initial PSA response) and during the entire observation period (best PSA response). Biochemical response is defined by the Prostate Cancer Working Group 3 (PCWG3) criteria as a PSA decline ≥ 50% 19. All clinical and hematological parameters (age, ALP, platelet and neutrophile count, hemoglobin and LDH) were assessed prior to first administration of the ^177^Lu-PSMA-617 therapy, during further therapy cycles, and during observation period and were analyzed according to their effect on survival.

### Stratification of the patient collective

Any PSA decline, a decline of ≥ 30 % and a decline of ≥ 50% were used to differentiate subgroups to evaluate the implications on OS (initial and best PSA response). Furthermore, patients were grouped according to whether they had received a second line chemotherapy with Cabazitaxel or not. In an additional approach, patients were grouped according to whether they presented with visceral metastases and compared with regards to OS.

### Statistics

SPSS Statistics 24 (IBM Corporation, Somers, NY, USA) and GraphPad Prism (version 7.0e, GraphPad, CA, USA) were used for statistical analysis. Descriptive statistics are reported as medians and IQR for continuous variables and frequencies for categorical variables. To identify parameters with significant impact on OS, multiple baseline and follow-up parameters were assessed by Kaplan-Meier estimates, log-rank tests and Cox proportional hazards ratios (HR) with corresponding 95% confidence interval (95%CI) in a univariate as well as multivariate analysis approach. Chi^2^-test was applied to test the relative distribution of visceral metastases between different treatment groups.

To detect significant differences, pairwise comparison of different groups was performed by students t-test using GraphPad Prism 7; *p*-values ≤ 0.05 are considered statistically noticeable.

## Results

Patient's characteristics are summarized in table 1. A total of 354 cycles of ^177^Lu-PSMA-617 with a median cumulative dose of 18.3 GBq (IQR 5.2 - 58.5) were applied to 109 patients. The median administered single dose was 6.2 GBq (IQR 5.8 - 6.48) with a median of three cycles ^177^Lu-PSMA-617 given (range one to nine cycles). Due to death or primary disease progression on ^177^Lu-PSMA-617, 21 patients (19%) received only one cycle of therapy.

At baseline, 93% of patients presented with bone metastases, 81% had lymph node metastases, and 44% showed visceral metastases. All patients were castration resistant. 85% had received at least one line of chemotherapy (Docetaxel) and 26% had been treated with a second-line chemotherapy with Cabazitaxel. All patients had received at least one line of next-generation anti-hormonal therapy with Abiraterone or Enzalutamide, 85% of whom had been treated with Enzalutamide and 83% with Abiraterone, 79% had received both. 16 patients had not been pretreated with Docetaxel due to contraindication for chemotherapy (e.g. heart failure, ECOG, age, etc.). In total, 54 patients (49%) died during the observation period.

**Figure 1:**
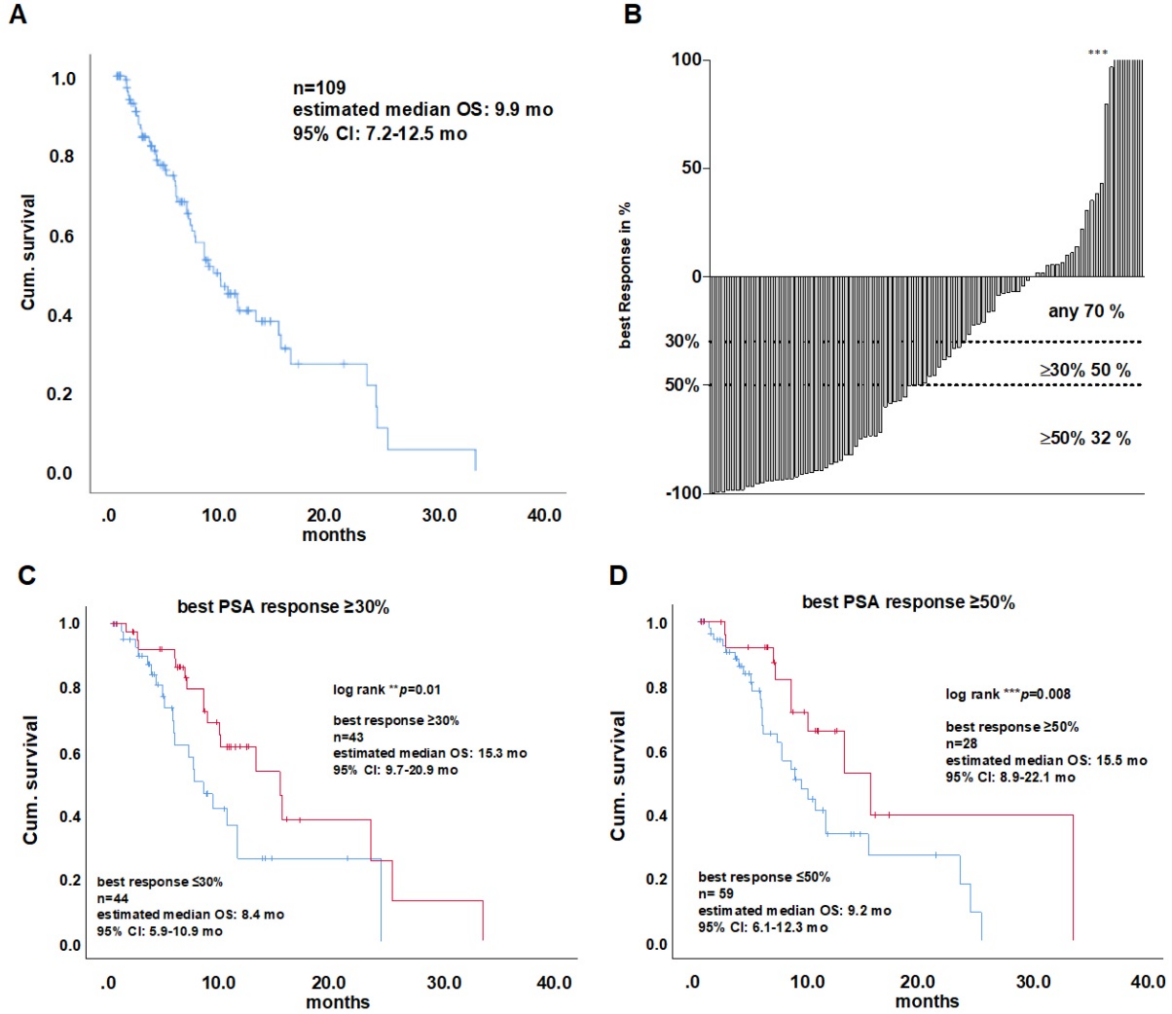
A) Kaplan-Meier plot of overall survival (OS) of the entire cohort. B) Waterfall plot of the percental PSA response distribution among all patients with a measurable PSA response. Response values, known critical for OS are indicated as threshold (dotted lines at 30, 50 and 90 %). Asterisks indicate values higher than 100 % PSA increase. Kaplan-Meier plots of overall survival of patients presenting a best PSA decline of ≥30% (C) and ≥50 %. (D). CI=confidence interval, PSA = prostate specific antigen

Kaplan-Meier analysis of the entire cohort revealed a median survival of 9.9 months (95%CI: 7.2 - 12.5, (Figure 1A). PSA response data of 87 patients were available for further analysis.

### Impact of blood parameters and PSA changes on overall survival

Any PSA decline occurred in 61 patients (70%), while 26 patients (30%) presented with PSA level increase. A proportion of 32% responded with a decrease ≥ 50%, 50% responded with a PSA decline ≥30%. Patients with any initial PSA decline after the first cycle of treatment showed a significantly longer OS (15.3 *vs.* 8.4 months, log rank test *p =* 0.01; HR 0.4, cox regression (CR) *p* = n.s.). An initial PSA decline of ≥50% was associated with prolonged survival (15.5 vs. 9.2 months, log rank test *p* = 0.031; HR 0.4, CR *p* = n.s.) as well as an initial decline of ≥30 % (15.3 vs. 8.4 months, log rank test *p =* 0.023; HR 0.4,CR *p* = n.s.). Like the initial PSA response rates, the analysis of best PSA response rates gave similar results with a decline of ≥30% (15.3 vs. 7.5 months, log rank test *p =* 0.01; HR 0.4, CR* p =* n.s.; Figure 1C). Best PSA decline ≥50% (Figure 1D) and any best PSA decline were also significantly associated with prolonged survival in Kaplan-Meier analysis (Log rank *p =* 0.008 and 0.03, respectively), but not after multivariate cox regression analysis.

There were no significant differences in OS between patients expressing ALP or LDH above or below the respective threshold (220 U/L and 240 U/L, respectively; Table 2).

### Pre-treatment and tumor-load-dependent survival

**Figure 2:**
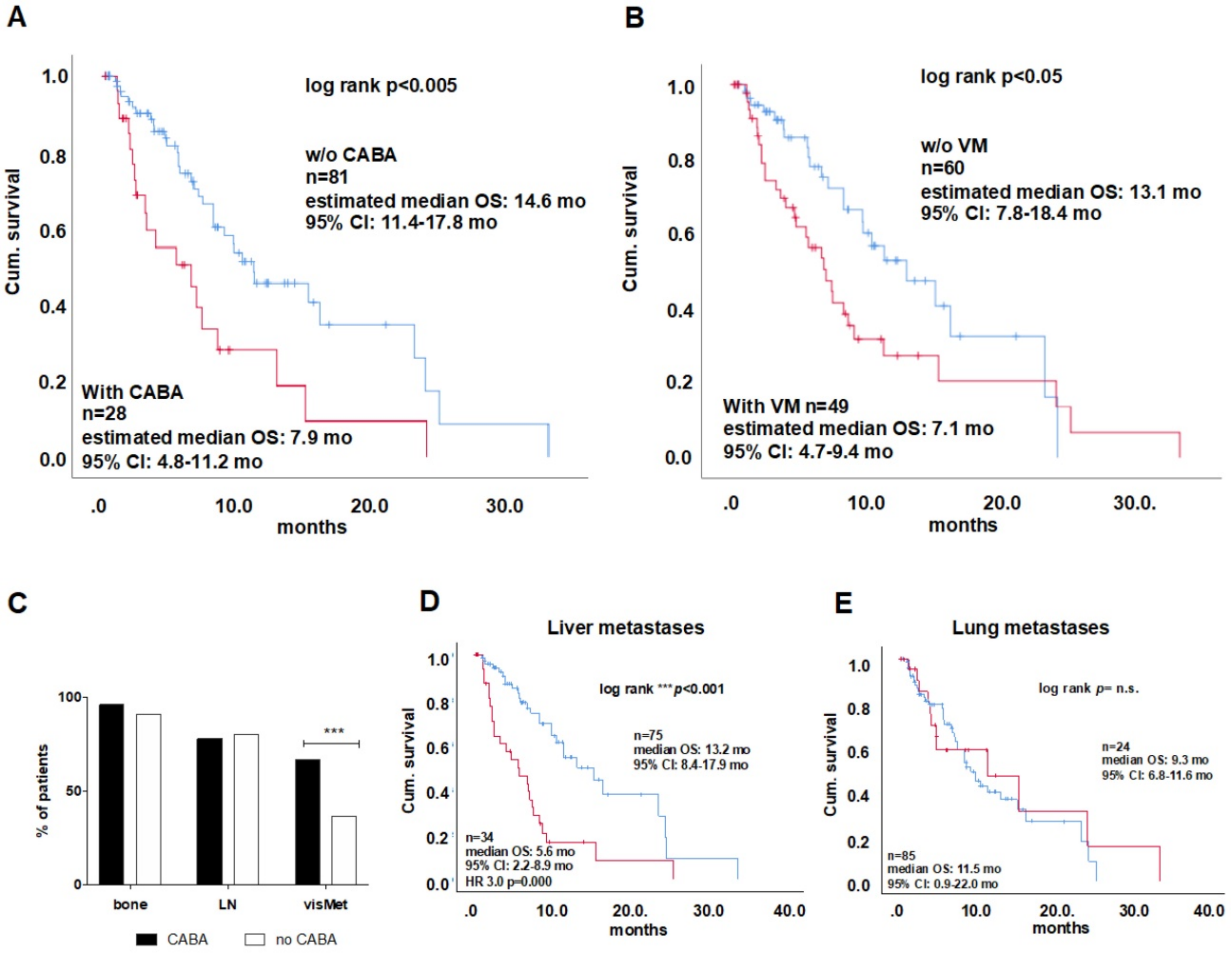
A) Kaplan-Meier plots of overall survival of patients who had or had not received Cabazitaxel treatment. Comparison of cabazitaxel treated patients vs. untreated. Kaplan-Meier curves for visceral metastases is shown in (B)The distribution of metastases among CABA-treated patinets is shown in a stacked column graph in %, result of Chi^2^-test for the distribution of visceral metastases between CABA and no CABA is indicated with asterisks***p<0.001 (C) Kaplan-Meier plots are shown for OS between patints with or without liver (D) and lung (E) metastases including log rank test results. CABA = Cabazitaxel; OS=overall survival; CI=confidence interval; LN = lymph node, visMet = visceral metastases, VM=visceral metastases.

The previous treatment with second line chemotherapy (Cabazitaxel) was identified to have an impact on OS: Twenty-eight patients (26%) had received second line Cabazitaxel, which was associated with shorter OS compared to patients who had not received second line chemotherapy (7.9 vs. 14.6 months, Log rank *p =* 0.002; HR 2.1, *p =* 0.009; Figure 2A).

Comparison of these two groups did not reveal any differences for Age, LDH, and ALP levels as well as baseline PSA and PSA response (Figure S1 A-E). Additionally, the metastatic patterns of patients pretreated with Cabazitaxel were different from those who had not received Cabazitaxel (Figure 2 C), with slightly higher numbers of bone metastases, but a higher rate of visceral metastases as confirmed by Chi^2^-test (67% vs. 37%; *p*<0.001; Figure 2C).

**Figure 3:**
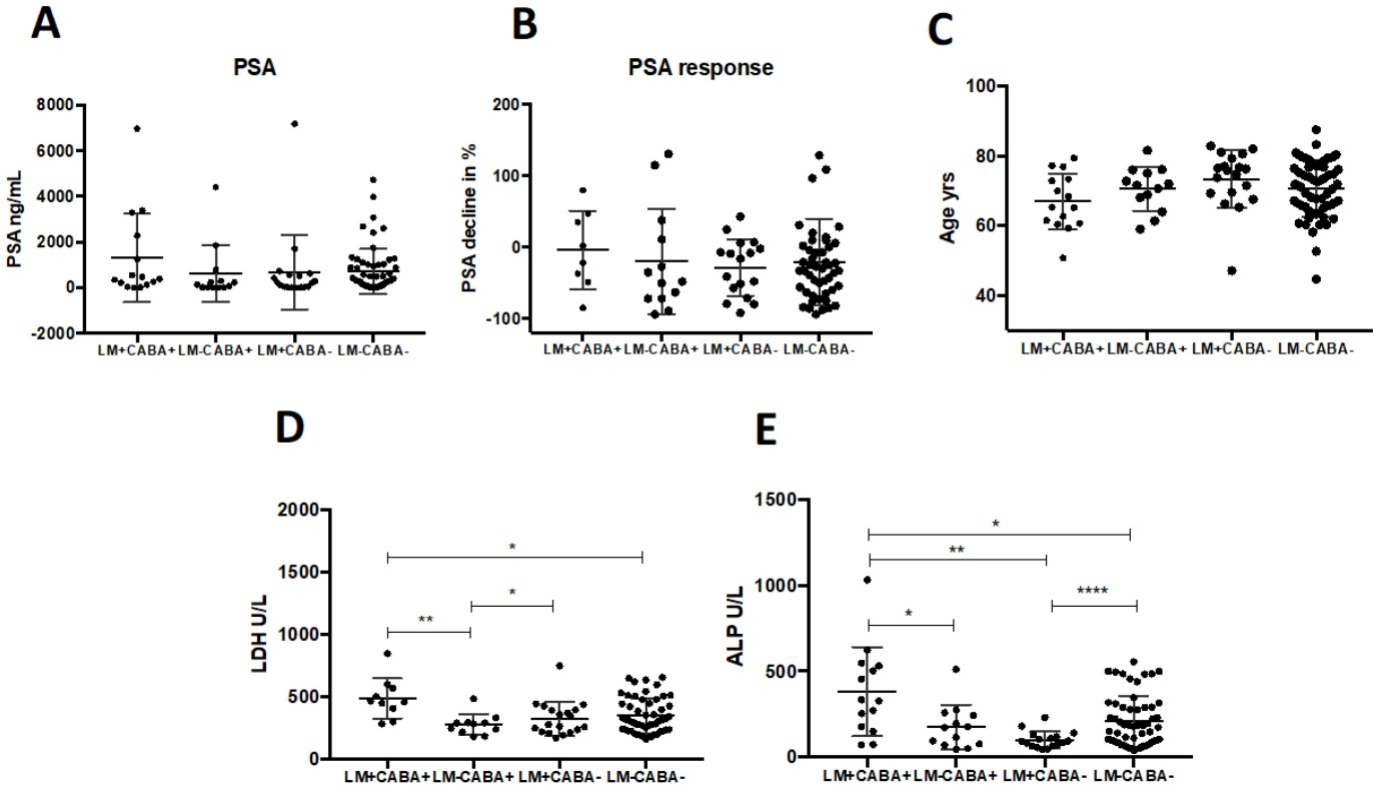
Dot plot comparisons are shown for certain parameters between four subgroups regarding liver metastases and cabazitaxel treatment including medians and standard deviation (A_E). Significant results are indicated by asterisks: *=p<0.01, **=p<0.001, ***=p<0.0001, ****=p<0.00001. PSA =prostate-specific antigen; LDH = lactate dehydrogenase; ALP = alkaline phosphatase; LM = liver metastases; CABA = cabazitaxel-treated

Fourty-eight patients (44%) of the entire cohort presented with visceral metastases, which was found to be associated with significantly shorter survival (7.1 *vs.* 13.1 months; log rank *p =* 0.029; Figure 2B) which remained as an independent predictor of OS (cox regression p<0.01, table 2). The highest impact on survival of all visceral metastases was observed for liver metastases (5.6 vs. 13.2 months, Log Rank: *p*<0.001; HR 3.0, *p*<0.001; Figure 2 D), while lung metastases had no significant influence on OS (9.3 vs. 11.5 months* p =* n.s.; Figure 2E). Patients pretreated with Cabazitaxel had a higher rate of liver metastases than patients without Cabazitaxel pretreatmtent (60 *vs.* 21%), while an equal proportion of each group had lung metastases (21 and 22%). To investigate whether Cabazitaxel (CABA) treatment and liver metastases (LM) have effects on survival, we performed an analysis with four groups: LM^+^CABA^+^ (n=15); LM^-^CABA^+^ (n=13), LM^+^CABA^-^ (n=19) and LM^-^CABA^-^ (n=61). Untreated patients (CABA-) without LM showed the longest OS (16.4 months; CI 95% 8.2-24.7), while CABA treated patients with LM (LM^+^CABA^+^) presented with the shortest OS in this comparison (3.3 months CI 95% 1.2-5.4; log rank *P*<0.001; Figure S2). OS did change with Cabazitaxel treatment, yet not on a significant level compared to untreated patients without LM (LM^-^CABA^-^ *vs.* LM^-^CABA^+,^ 16.4 vs. 13.2 months; Figure S1B). On the other hand, OS of untreated patients with LM was significantly shorter than OS of LM^-^CABA^-^ -patients (16.4 vs. 6.9 months; CI95% 8.2-24.7 vs. 4.7-9.1, respectively; Figure S2C). Noticeably, the effects of LM and CABA treatment seem to add up and shorten life expectation significantly (16.4; CI 8.2-24.7 vs. 3.3; CI 1.2-5.4 months; log rank *p*<0.001). There were no significant differences regarding PSA level or PSA response (Figure 3 A-C), nor in any other marker or condition that was compared, except the parameters LDH and ALP. LDH levels were significantly elevated in LM^+^CABA^+^ patients compared to the remaining three groups (Figure 3D+E). Yet, CABA treatment seems to have more impact on LDH serum levels than LM, while LM seem to influence ALP serum levels stronger than CABA treatment.

Further, the effect of external beam radiation therapy (EBRT) as well as next-generation anti- hormonal therapy and Radium-223on OS was investigated and was not significant (data not shown).

## Discussion

The present study investigates the role of prior treatment strategies and biochemical parameters, such as PSA, ALP and LDH in 109 patients with mCRPC undergoing ^177^Lu-PSMA-617 RLT and their impact on OS.

Recent studies have postulated that a PSA response of ≥50% is a critical prognostic factor for OS in patients with mCRPC receiving ^177^Lu-PSMA-617 therapy 6, 15, 20.

As shown in an earlier study, the present study confirmed that a decline in PSA levels of ≥50%, defined as biochemical response by PCSWG3, was associated with prolonged survival 17. Furthermore, a PSA decline ≥30% was identified to be an equally powerful prognosticator in the present study. This is in line with another previous trial that showed that the optimal PSA-decline-cut-off under ^177^Lu-PSMA- 617 RLT was 20.87% 15. As described, any PSA decline may be a useful marker for therapy response in ^177^Lu-PSMA-617 RLT 9, whereas a decline of ≥50% or ≥30% may be more reliable, as recently described by Rahbar et al. and Heck et al. 15, 20

The positive effects of Cabazitaxel after other chemotherapeutics, such as Docetaxel, has been investigated in other studies with positive outcomes, such as improvement of health-related quality of life and pain control 21. However, no study so far investigated the long-term effects of Cabazitaxel treatment with regards to the efficacy of subsequent ^177^Lu-PSMA-617 RLT. In the present cohort, patients treated with ^177^Lu-PSMA-617 RLT showed a shorter survival when having been pretreated with Cabazitaxel, compared to untreated individuals. The two groups did not vary with regard to PSA at baseline, and PSA response as well as age, ALP and LDH levels at baseline. Furthermore, similar levels of hemoglobin, age, platelets and neutrophil counts, as well as similar performance status of the patients (ECOG and Karnofsky scores (data not shown) were observed. However, noticeably, the metastatic burden was distinctively different in the patients that had been treated with cabazitaxel before. Here, almost twice as many patients had visceral metastasis compared to those patients who were cabazitaxel- naïve. Since chemotherapy is effective in visceral, especially liver metastasis, this may have been part of the reason why cabazitaxel had been given previously. And, despite second line chemotherapy may reduce the tumor burden, however, the remaining metastases may act more aggressively, as indicated by disease progression and earlier death. This might be due to tumor heterogeneity, causing a response of differentiated tumor lesions to therapy, whereas the more aggressive tumor lesions do not respond at all, possibly due to a cabazitaxel resistance. There is evidence that cancer cells of soft tissue metastases such as those sited in the liver might express more survivin and, hence, poorly or not at all respond to apoptosis inducing therapies 22. Further, resistance to taxanes, such as cabazitaxel is reported as a multi-drug resistance accompanied by elevated class III β-tubulin RNA (TUBB3) expression and altered microtubule dynamicity, as well as decreased expression of the cell cycle regulator BRCA1 and the induction of epithelial-to-mesenchymal transition (EMT) in breast and ovarian cancer models 23. Another study using soft tissue metastasis-derived mCRPC cell lines resistant to cabazitaxel, suggest elevated ERK and PI3K/AKT signaling as crucial for cabazitaxel resistance in mCRPC 24. However, the details of the underlying mechanisms for this resistance in prostate cancer in particular remain unclear and definitely call for a detailed prospective investigation. Yet, it remains to be discussed, if cabazitaxel resistance is particular for soft tissue metastases, and if absent, PSMA expression is a result of its heterogeneity among circulating tumor cells that colonize soft tissue for metastasis formation in patients with end stage mCRPC 25. The presence of visceral metastases was found critical for survival in previous studies and in this cohort, whereas liver metastases seem to have more impact than lung metastases 15. In detail, a larger proportion of patients treated with cabazitaxel presented with visceral metastases (67%) compared to patients without (37%). Additionally, high tumor volume has been described as a risk factor for worse survival 26. In this study liver metastases and cabazitaxel treatment have been identified as independent risk factors for survival. While for CABA treatment we only observe a trend in reduction of OS, it is significantly reduced by the presence of liver metastases. In CABA treated patients with liver metastases, however the effects of both risk factors seem to add up and shorten life expectation tremendously. This effect can be expected to be even more pronounced with a longer observation period, as 80% of LM^+^CABA^+^ and 73% of LM^+^CABA^-^ patients already had died during the observation period, while 46% and 35% of LM^-^CABA^+^ and LM^-^CABA^-^ patients, respectively were still alive at termination of the observation. To face the mortal risk of liver metastases that are low or potentially void of PSMA expression, we propose the investigation of a combined or alternating therapy using ^177^Lu-PSMA-617 RLT and local liver targeted therapy e.g. selective internal radiotherapy (SIRT) or external radiation 27. This treatment strategy would target PSMA-positive lesions in bone and soft tissues as well as PSMA-negative liver metastases that are critical for OS.

This study faces a number of limitations due to its retrospective design. Retrospective data collection may have led to incomplete records of possible confounders and therefore overestimation of the reported effects. Moreover, patients in this study have not been selected randomly, but retrospectively enrolled because they fulfill certain predesigned criteria. However, these data have been collected without any hypothesis and purpose which should exclude any systematic bias.

## Conclusions

^177^Lu-PSMA-617 is an effective therapy for patients with mCRPC. However, the present data indicate that its beneficial effects on OS are strongly influenced by pretreatment therapeutic strategy (history of second line chemotherapy with cabazitaxel) and the presence of visceral metastases at onset of ^177^Lu-PSMA-617 treatment. The latter remained the only independent predictor of OS. This warrant focusing on better understanding of sequencing therapies in mCRPC and focal treatment of liver metastases in future studies. Further prospective controlled trials have to confirm these data.

## Figures and Tables

**Table 1:** Patient baseline characteristics

Parameters	Median	(IQR)
Age	72	(44.7-87.5)
Gleason score	8	(7.8-8.4)
PSA (ng/ml)	294	(2.74-7190)
**ECOG PS**	**n**	**%**
0-1	85	77.3
2	21	19.1
unknown	4	3.6
Alkaline phosphatase (U/l)	173	(38-1033)
LDH (U/l)	326	(160-7802)
**Site of metastases**	**n**	**%**
Bone	102	93.5
Lymph node	87	79.8
Liver	34	31.2
Lung	24	22.0
other	2	1.8
**Previous therapy of mCRPC**	**109**	**100%**
Docetaxel	93	85.3
Cabazitaxel	28	25.7
Abiraterone	91	83.4
Enzalutamide	93	85.3
Both (Abiraterone & Enzalutamide)	86	78.9
^223^Radium	22	20.1
External beam radiation to bone	47	43.1

PSA = Prostate-specific antigen; ECOG PS = Eastern Co-operative Oncology Group Performance Status; LDH = Lactate Dehydrogenase; mCRPC = metastatic Castration-Resistant Prostate Cancer.

**Table 2:** Univariate and multivariate analysis of overall survival.

Subgroup	Patients (n)	Events (n)	mOS (months)	Hazard ratio (95% CI)	*P*_Log rank_	*P*_cox regression_
**Any initial PSA decline**						
Yes	61	28	15.3	0.4 (0.2-0.8)	0.01	n.s.
No	26	11	8.4	1 (reference)		
**Initial PSA decline ≥50%**						
Yes	28	10	15.5	0.4 (0.2-0.9)		
No	59	29	9.2	1 (reference)	0.03	n.s.
**Initial PSA decline ≥30%**						
Yes	43	18	15.3	0.4 (0.2-0.9)	0.03	n.s.
no	44	21	8.4	1 (reference)		
**baseline Alkaline phosphatase**						
<220 U/L	67	32	9.8	0.3 (0.6-1.7)	n.s.	n.s.
>220 U/L	42	22	9.2	1 (reference)		
**baseline LDH**						
<240 U/L	33	17	9.8	0.9 (0.6-1.7)	n.s.	n.s.
≥240 U/L	76	37	9.2	1 (reference)		
**Visceral metastasis**						
Yes	49	32	6.9	2.0 (1.2-3.6)	0.029	**0.006**
No	59	21	15.3	1 (reference)		
**Second line chemotherapy (Cabazitaxel)**						
Yes	28	18	6.8	2.1 (1.2-3.8)		
No	81	36	11.5	1 (reference)	0.005	n.s.
**Any best PSA decline**						
Yes	65	29	15.3	0.4 (0.2-0.8)	0.03	n.s.
No	23	12	8.4	1 (reference)		
**Best PSA decline ≥50%**						
Yes	41	16	15.3	0.4 (0.2-0.8)		
No	47	25	7.5	1 (reference)	0.008	n.s.
**Best PSA decline ≥30%**						
Yes	51	22	15.3	0.4 (0.2-0.9)	0.01	n.s.
no	37	19	7.5	1 (reference)		

PSA = Prostate-specific antigen; LDH = Lactate Dehydrogenase, *p*-values that remained significant after multivariate analysis are shown in bold.

## Abbreviations

ALP: alkaline phosphatase
ECOG: Eastern Co-operative Oncology Group
LDH: lactate dehydrogenase
mCRPC: metastatic castration-resistant prostate cancer
PSMA: prostate-specific membrane antigen
PSA: prostate-specific antigen
RLT: radioligand therapy
