# Supplementary material for: Second line chemotherapy and visceral metastases are associated with poor survival in patients with mCRPC receiving 177Lu-PSMA-617
Source: Theranostics. 2019 Jul 9;9(17):4841–8. doi: 10.7150/thno.35759 (PMC6691377; doi:10.7150/thno.35759)
Supplement: Supplementary file 1 — Supplementary figures and tables. [file thnov09p4841s1.pdf]

Supplemental Material

Supplementary table 1: patient characteristics of subgroups regarding liver metastases and cabazitaxel treatment

**Parameters**	**LM+ CABA+**	**IQR**	**LM- CABA+**	**IQR**	**LM+ CABA-**	**IQR**	**LM- CABA-**	**IQR**
Number of patients	15		13		19		62	
Age	65.4	(60.7-73.4)	71.6	(66.0-7.5)	75.9	(69.3-79.3)	71.9	(65.3-77.6)
Gleason score	8	(8-9)	8	(7-9)	8	(7-9)	8	(7-9)
ECOG	0	(0-2)	1	(0.25-1)	1	(0-1)	1	(0-1)
PSA (ng/ml)	378.9	(128.8-2282.0)	132.0	(13.1-542.5)	208.4	(29.9-561.4)	329.6	(93.4-977.0)
ALP (U/l)	330.5	(171-534.5)	172.0	(73.5-250.5)	94.0	(66.0-141.0)	185.0	(89.3-315.8)
LDH (U/l)	484.0	(418.3-783.8	282.0	(218.0-294.0)	277.0	(219.0-396.0)	326.0	(236.0-510.0)
**Site of metastases**	**Total**	**%**	**Total**	**%**	**Total**	**%**	**Total**	**%**
Bone	15	100	12	92	17	89	55	88
Lymph node	13	87	8	62	15	79	50	80
Lung	4	27	2	15	5	26	12	29
other	0	0	1	8	1	5	0	0
**Previous therapy of mCRPC**	**Total**	**%**	**Total**	**%**	**Total**	**%**	**Total**	**%**
Docetaxel	15	100	13	100	15	79	48	77
Abiraterone	13	87	12	92	15	79	50	80
Enzalutamide	11	73	13	100	17	89	51	82
Both (Abiraterone & Enzalutamide)	11	73	12	92	13	68	42	68
^{223}Radium	3	20	2	15	3	16	14	23
EBRT-bone	3	20	3	20	10	53	10	16

LM=liver metastases; CABA=cabazitaxel treatment; PSA=prostate-specific antigen; ALP=alkaline phosphatase; LDH=lactate dehydrogenase; EBRT=external beam radiation; IQR=interquartile range

Supplementary table 2: Survival parameters of patient subgroups regarding liver metastases and cabazitaxel treatment

Parameters	LM+ CABA+	CI (95%)	LM- CABA+	CI (95%)	LM+ CABA-	CI (95%)	LM- CABA-	CI (95%)
Number of patients	15		13		19		62	
Deaths during observation	12	80%	6	46%	14	73%	22	35%
Median OS	3.3	(1.2-5.4)	13.2	(0.8-25.6)	6.9	(4.7-9.1)	16.4	(8.2-24.7)
Median PFS								

LM=liver metastases; CABA=cabazitaxel treatment; PSA=prostate-specific antigen; ALP=alkaline phosphatase; LDH=lactate dehydrogenase; EBRT=external beam radiation; CI = confidence interval; PFS = progression free survival (PCSWG3 criteria, ≥25% and ≥2ng/ml of nadir)

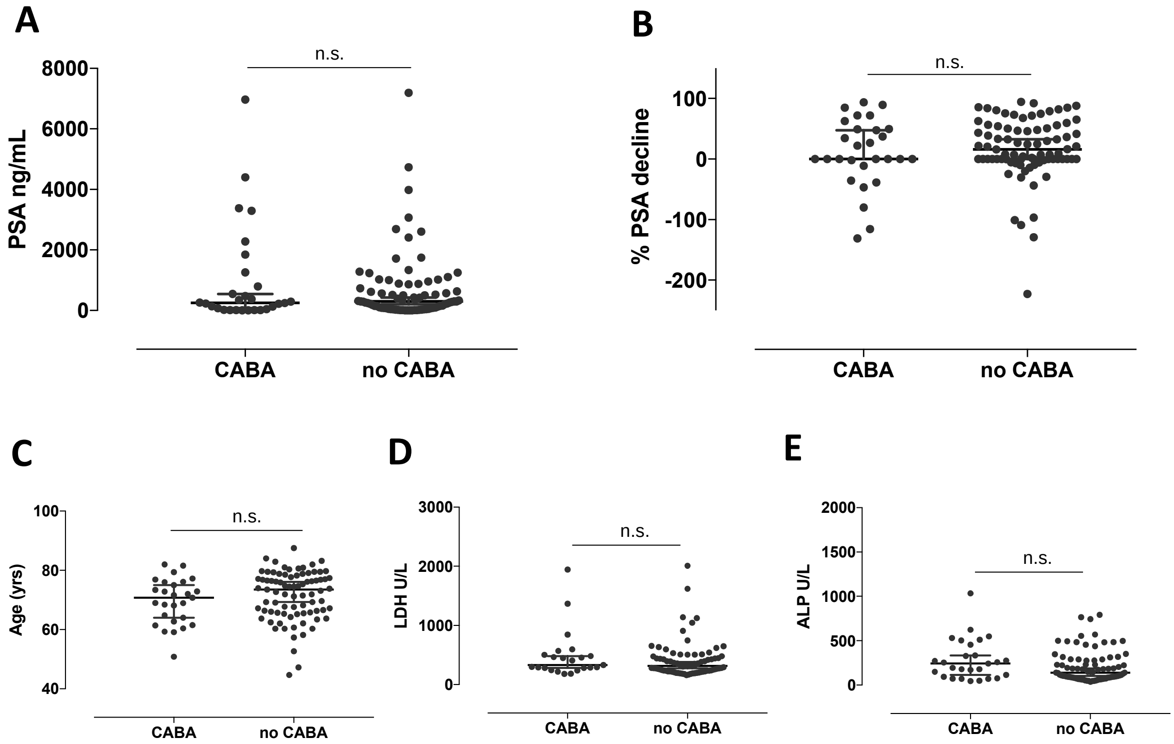

Figure S1: comparison of CABA-treated and untreated patients regarding different parameters. Dot plot comparisons are shown for PSA level (A) PSA response (B), age (C), LDH (D) and ALP (E). both groups have been. Compared using a non-parametric t-test. PSA = prostate-specific antigen, LDH = lactate dehydrogenase; ALP = alkaline phosphatase; CABA = cabazitaxel; n.s. = not significant

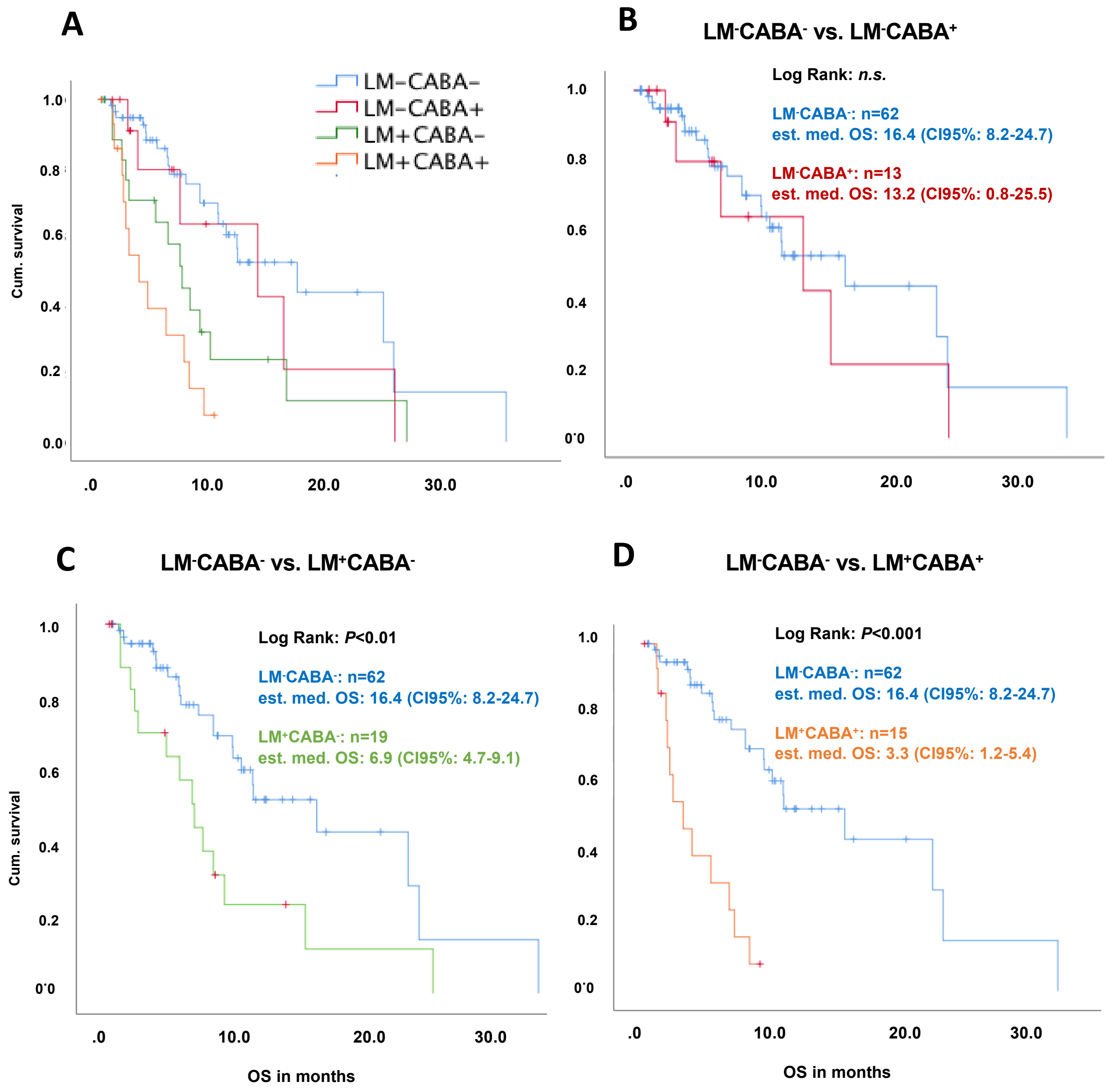

Figure S2: Kaplan-Meier survival analysis of four subgroups $LM^{+}CABA^{+}$ (n=15); $LM^{-}CABA^{+}$ (n=13), $LM^{+}CABA^{-}$ (n=19) and $LM^{-}CABA^{-}$ (n=62) (A). Individual comparisons of OS of $LM^{-}CABA^{+}$ (B), $LM^{+}CABA^{-}$ (C) and $LM^{+}CABA^{+}$ (D) with $LM^{-}CABA^{-}$. LM = liver metastases, CABA = Cabazitaxel, OS = overall survival, cum. survival = cumulative survival
